# Skin Tumors among Biopsy Samples in Patients Attending Dermatological Out Patient Department in a Tertiary Care Hospital of Nepal: A Descriptive Cross-sectional Study

**DOI:** 10.31729/jnma.6955

**Published:** 2021-09-30

**Authors:** Shristi Shrestha, Arnija Rana, Deepika Karki, Asim Shrestha

**Affiliations:** 1 Department of Dermatology, Nepal Medical College and Teaching Hospital, Jorpati, Kathmandu, Nepal; 2 Nepal Medical College and Teaching Hospital, Jorpati, Kathmandu, Nepal

**Keywords:** *benign*, *malignant*, *skin neoplasms*, *tumors*

## Abstract

**Introduction:**

Skin tumors are on the rise in the Nepalese community. The different morphological pattern of skin tumors requires its meticulous categorization for understanding its effect on prognosis and treatment. Our study aimed at studying the prevalence of skin tumors among the skin biopsies performed in the dermatology outpatient department in a tertiary care hospital of Nepal.

**Methods:**

A descriptive cross-sectional study was done from skin biopsy samples from 1^st^ January, 2017 to 31^st^ December, 2019, at a tertiary care center. Ethical clearance was taken from the institutional review committee (IRC), Ref No: 056-077/078. Convenience sampling was done. A self-designed proforma containing questions on the patients' socio-demographic data and clinical details were used, and a biopsy of those clinically suspected to have skin tumors was done. Skin tumors were classified according to the World Health Organization 2018 classification of skin tumors. Data were analyzed using Statistical Package for the Social Sciences Version 16. Point estimate at 95% Confidence Interval was done, and frequency and proportion were calculated.

**Results:**

A total of 671 skin biopsies were done during this study, out of which 125 (18.63%) at 95% Confidence Interval (15.68-21.57) were diagnosed with skin tumors. Among them, 77 (61.6%) were female, and 48 (38.4%) were male. Among the diagnosed cases, 105 (84%) were benign, and 20 (16%) were malignant. Females showed preponderance in both benign and malignant tumors.

**Conclusions:**

The findings from our study show the increasing prevalence of skin tumors, and the results were comparable to other similar studies conducted in various parts of Nepal.

## INTRODUCTION

Skin tumors result in a proliferation of various components of the skin. They can be benign, simply causing cosmetic concern to premalignant lesions and aggressive tumors.^1^ Benign tumor are circumscribed, have symmetrical architecture, uniform cell nuclei, restraint rate of growth, and absence of metastases.^2^ Malignant tumors are poorly circumscribed, have less symmetrical architecture, atypical cell nuclei, rapid growth, and potential to give rise to metastases.^2^

In Nepal, a study found cutaneous infections and dermatitis to account for the majority of cases.^3^ The indolent 'lumps' and 'bumps' are seen less frequently seeking medical advice.^1^ These tumors have an extensive histo-morphological pattern, and various names are used to denote the same tumors.^4^ So, it is challenging to study their comprehensive types.

Though skin tumors are rare in our population, they opt for investigations to confirm it with the increasing awareness among patients. Our study aimed at studying the prevalence of skin tumors and their types among the skin biopsies performed in the dermatology outpatient department in a tertiary care hospital of Nepal.

## METHODS

This study was a descriptive cross-sectional study conducted in Nepal Medical College and Teaching Hospital among the patients visiting dermatological OPD from 1^st^ January, 2017 to 31^st^ December, 2019. Ethical clearance was approved from the Institutional Review Committee (IRC) of the college, (Reference number: 056-077/078).

All the patients who were suspected to have skin tumors on the basis of history and clinical examination were asked to get a skin biopsy done and those who consented to get a biopsy done were included in the study. In the first step, a self-designed proforma containing questions on socio-demographic data and clinical details of the patients was administered then depending on the type of lesion punch biopsy, excisional biopsy, incisional biopsy and shave biopsy was done. The histopathological diagnosis was then considered to be the final diagnosis and the confirmed tumor cases were further classified according to World Health Organization (WHO) 2018 classification of skin tumors.^5^

Convenience sampling was done and minimum sample size was calculated as follows:

n = Z^2^ × (p × q) / e^2^

  = (1.96)^2^ × (0.197 × 0.803) / (0.07)^2^

  = 124

Where,

n = minimum sample size
Z = 1.96 at at 95% Confidence Interval (CI)
p = prevalence, 19.7%^8^
q = (1-p)
e = margin of error, 7%

Selection bias has been minimized as possible and collected data was analyzed using SPSS 16 following which descriptive analysis was done. Point estimate at 95% Confidence Interval was done for binary data along with analysis for frequency and proportion.

## RESULTS

A total of 671 skin biopsies were done during this period and 125 were confirmed to be skin tumors on histopathological examination, the frequency being 18.63% out of the total biopsied sample. Out of 125 patients, 20 (16%) had malignant tumors, whereas 105 (84%) had benign tumors.

When the skin tumors were classified according to WHO 2018 classification, majority were keratinocytic 44 (35.2%), followed by soft tissue tumors 39 (31.2%), melanocytic tumors 21 (16.8%), appendageal tumors 12 (9.6%) and neural tumors 9 (7.2%). Among benign tumors, the majority were soft tissue tumors 39 (37.1%) followed by keratinocytic tumors 26 (24.7%), melanocytic 20 (19.1%), appendageal 11 (10.5%) and neural tumors 9 (8.6%). Whereas, among malignant tumors, keratinocytic tumors were the commonest in 18 out of 20 (90%). Appendageal tumors and melanoma were seen in 1 patient each. Malignancy of soft tissue was not seen at all (Table 1).

**Table 1:** Distribution of skin tumors according to WHO classification 2018.

Group of neoplasm	Benign tumor n (%)	Malignant tumor n (%)	Total n (%)
Keratinocytic tumor	26 (20.8)	18 (14.4)	44 (35.2)
Appendageal tumor	11 (8.8)	1 (0.8)	12 (9.6)
Melanocytic tumor	20 (16.0)	1 (0.8)	21 (16.8)
Soft tissue tumor	39 (31.2)	0 (0.0)	39 (31.2)
Neural tumor	9 (7.2)	0 (0.0)	9 (7.2)
Total	105 (84.0)	20 (16.0)	125 (100)

The age group of patients ranged from 45 days to 90 years of age and the maximum number of patients were in the age group of 31-40 years.

Benign tumors were observed more in the age group of 31-40 years. Benign soft tissue tumors were observed mostly in the age group of 31-40 years, whereas keratinocytic benign tumors were more frequent in 5160 years of age. Malignant tumors were observed more frequently in the older age groups of 51-60 years and 71-80 years (Table 2). Females showed preponderance in both benign and malignant skin tumors (Table 3).

**Table 2:** Distribution of skin tumors (benign and malignant) in various age groups.

	Keratinocytic tumors		Appendageal tumors		Melanocytic Mel­anoma		Soft tissue tumors		Neural tumors		Total n (%)
	Benign n (%)	Malignant n (%)	Benign n (%)	Malignant n (%)	Benign n (%)	Malignant n (%)	Benign n (%)	Malignant n (%)	Benign n (%)	Malignant n (%)	
0-10yrs	0	0	0	0	0	0	3 (7.69)	0	0	0	3 (2.4)
11-20yrs	2 (7.69)	0	3 (20)	0	4 (20)	0	5 (12.82)	0	1 (11.11)	0	15(12%)
21-30yrs	1 (3.84)	0	2 (10)	0	5 (25)	0	10 (25.64)	0	2 (22.22)	0	20 (16)
31-40yrs	5 (19.23)	1 (5.55)	4 (13)	0	6 (30)	0	12 (30.77)	0	3 (33.33)	0	31 (24.8)
41-50yrs	5 (19.23)	2 (11.11)	0	0	2 (10)	0	0	0	2 (22.22)	0	11 (8.8)
51-60yrs	8 (30.77)	4 (22.22)	0	1 (100)	2 (10)	1 (100)	6 (15.38)	0	0	0	22 (17.6)
61-70yrs	3 (11.54)	4 (22.22)	1 (8.33)	0	1 (5)	0	3 (7.69)	0	0	0	12 (9.6)
71-80yrs	1 (3.84)	6 (33.33)	1 (11.11)	0	0	0	0	0	1 (11.11)	0	9 (7.2)
81-90yrs	1 (3.84)	1 (5.55)	0	0	0	0	0	0	0	0	2 (1.6)
Total	26 (100)	18(100)	11 (100)	1 (100)	20 (100)	1 (100)	39 (100)	0	9 (100)	0	125 (100)

**Table 3:** Gender wise distribution of benign and malignant tumors.

Sex	Benign	Malignant	Total n(%)
	Number of patients n (%)	Number of patients n (%)	
Female	61 (58.1)	16 (80)	77 (61.6)
Male	44 (41.9)	4 (20)	48(38.4)
Total	105 (100)	20 (100)	125(100)

The commonest site involved was the head, neck and face region in 60 (48%) patients. Other sites of involvement were trunk 31 (24.8%), upper limb 17 (13.6%), lower limb 14 (11.2%) and external genitalia 3 (2.4%).

Among the 12 appendageal tumors, benign tumors were 11 in number and only one was malignant tumor which was extra mammary Paget's disease. Among benign appendageal tumors, the commonest was tumor with follicular differentiation in 8 (72.7 %) followed by tumors with eccrine and apocrine differentiation in 3 (27.3 %) (Table 4).

**Table 4:** Distribution of skin appendageal tumors according to WHO classification 2018.

Appendageal Tumors	Benign n (%)	Malignant n (%)	Total n (%)
Tumor with Follicular differentiation	8 (72.7)	0 (0.0)	8 (66.7)
Tumor with eccrine and Apocrine differentiation	3 (27.3)	0 (0.0)	3 (25)
Site specific tumor: Extramammary Paget disease	0	1 (100)	1 (8.3)
Total	11 (100)	1 (100)	12 (100)

Out of 105 benign tumors, seborrheic keratosis was the most frequently observed in 20 (19%) followed by intradermal nevus in 14 (13.3%). Other frequently encountered benign tumors were dermatofibromas in 11 (10.4%) and lobular capillary hemangiomas in 11 (10.4%) followed by neurofibromas in 8 (7.6%) (Table 5). Among malignant keratinocytic tumors, Basal cell carcinoma (BCC) was the commonest in 55% (11 out of 20), followed by Squamous cell carcinoma (SCC) in 25% (5 out of 20) (Table 5).

**Table 5:** Frequency of tumors of skin according to WHO 2018 classification.

S.N	Variables	n (%)
	Benign Tumors	Number of patients n (%)
**1**	**Benign Keratinocytic tumors**	
	Squamous papilloma	2 (1.9)
	Seborrheic keratosis	20 (19.0)
	Stucco keratosis	1 (0.9)
	Verruca vulgaris	3 (2.9)
**2**	**Appendageal tumors**	
**Follicular differentiation**		
	Trichoblastoma	3(2.9)
	Pilomatricoma	3(2.9)
	Trichofolliculoma	2 (1.9)
**Eccrine and Apocrine differentiation**		
	Syringoma	1 (0.9)
	Spiradenoma	1 (0.9)
	Syringocystadenoma papilliferum	1 (0.9)
**4**	**Melanocytic tumors**	
	Lentiginous junctional melanocytic nevus	1 (0.9)
	Junctional nevus	1 (0.9)
	Compound nevus	3(2.9)
	Intradermal naevus	14 (13.3)
	Pigmented spindle cell nevus	1 (0.9)
**5**	**Soft tissue tumors**	
Adipocytic tumors		
	Lipoma	5 (4.7)
	Angiolipoma	1 (0.9)
Fibroblastic and fibrohistiocytic tumors		
	Dermatofibroma	11 (10.4)
	Fibromas	3(2.9)
	Cutaneous myxoma	1 (0.9)
**Smooth muscle tumors**		
	Cutaneous leiomyoma	2 (1.9)
**(Myo)pericytic tumors**		
	Glomus tumor	1 (0.9)
**6**	**Vascular tumors Hemangiomas**	
	Epithelioid hemangioma	1 (0.9)
	Angiokeratoma	2 (1.9)
	Infantile hemangioma	1 (0.9)
	Lobular capillary hemangioma	11 (10.4)
**Neural tumors**		
	Neurofibroma	8(7.6)
	Schwannoma	1 (0.9)
	Total	105 (100)
**Malignant tumors**		
**1**	**Keratinocytic tumors**	
	Basal cell carcinoma	11 (55.0)
	Squamous cell carcinoma	1 (35.0)
**2**	**Appendageal tumors**	
	Site-specific tumor Extramammary Paget disease	1 (5.0)
**3**	**Melanocytic tumor Desmoplastic melanoma**	1 (5.0)
	**Total**	20 (100)

Both of these malignancies were more frequently observed among females with basal cell carcinoma in 9 (45%) females; squamous cell carcinoma in 6 (30%); extramammary Paget disease in 1 (5%); and desmoplastic melanoma in 1 (5%) females.

## DISCUSSION

The incidence of skin cancer is increasing globally.^6^ Various studies from Nepal has also shown an increasing trend in skin cancers.^7,8^ Skin biopsy is a simple and inexpensive procedure, which identifies the architectural pattern of tumor and growth of infiltration to distinguish the malignant tumour.^9,10^ It is important to diagnose the malignant skin tumors due to its therapeutic implications and aggressive nature.^1,9^ Management of benign tumors are usually done for cosmetic reasons.^1^

Various studies have shown that benign tumors are common in younger age groups whereas malignant tumors are mostly seen in older age groups.^9,19^ A similar pattern was noted in our study with maximum number of benign neoplasms in 31-40 years and malignant tumors in 51-60 years and 71-80 years. Genetic susceptibility, environmental factors, mainly chronic exposure to ultraviolet (UV) radiation, ionizing radiation, and chemical carcinogens such as arsenic exposure are some of the risk factors for non-melanoma skin carcinoma.^20-24^

Our study showed a female preponderance among both the benign and malignant tumors. Females also had more benign tumors in various other studies from Nepal.^10-12^ Similar to our study malignant skin tumors were commoner in females in a study by Adhikari et al.^19^ This may be due to the fact that females have health seeking behavior for their skin problems as they are more cosmetically concerned compared to males.

In our study, maximum number of skin tumors, both benign and malignant were seen in the head, face and neck region which is comparable with the studies from Karki et al, Shrivastava et al and Narhire et al.^10,13-14^ Head, face and neck are the sun exposed areas and of cosmetic concern to the patients, which might explain the high frequency of tumors.

Majority of skin tumors are benign in nature.^1^ Similarly, in our study as well out of 125 skin tumors, 84% were benign and 16% were malignant. In the study done by Sherpa and KC^11^, out of 214 skin tumors, 81.8% were benign and 18.2% were malignant.^11^ Similar results were obtained from various studies from Nepal.^10,12^ Studies from India also showed higher number of benign tumors.^9,13-14^

In our study, the majority of skin tumors were keratinocytic 35.2%, followed by soft tissue tumors 31.2%, melanocytic tumors 16.8% and appendageal tumors 9.6%. Keratinocytic tumors have been observed to be the commonest skin tumors in Nepal in studies done by Sherpa and KC and Thapa et al also.^11,12^ Maximum number of malignant tumors in our study belonged to the keratinocytic category by 90%. Similar findings were observed in various studies.^10,11-12^

In our study, out of 105 benign tumors, soft tissue tumors of dermis (37.1%) were commonest followed by keratinocytic tumors. Karki et al also showed out of 142 cases of benign tumors, soft tissue tumors of the dermis (34%) were commonest followed by keratinocytic tumors.^10^ Some studies found appendageal tumors to be more frequent among the benign skin tumors.^13,15^

Out of 20 malignant tumors in the study, BCC was the commonest in 11 cases (55%) followed by SCC which were in 5 cases (25%). Similar results of higher frequency of BCC have been reported in studies done in Nepal as well as other countries.^7,16-19^ But some other studies have shown SCC as the commonest malignant skin tumor.^9-15.^

Malignant melanoma was found to be the least common malignant tumor constituting only a single case in this study. Similar results of less frequency of melanoma were seen in various other studies from Asia.^11-13,16-18^ This is in contrast to the higher frequencies of melanoma from Australia and European countries.^25-27^

In the present study, among the benign tumors, seborrheic keratosis was the most common 19.0%, followed by intradermal nevus 13.3%. Seborrheic keratosis was the second most common in the study by Thapa et al.^12^

Appendageal tumors comprised 9.6% of total cases, out of which benign appendageal tumors 10.5% were more common than malignant 5%. Karki et al, also showed similar results of higher frequency of benign appendageal tumors compared to malignant.^10^ Tumors with follicular differentiation 72.7% were frequently encountered among the benign appendageal tumors. This was followed by tumors with eccrine apocrine differentiation in 37.3%. In our study, pilomatricoma, 2.9% and trichoblastoma, 2.9% were the commonest among follicular benign tumors. Pilomatricoma was also the commonest appendageal tumors in various other studies.^11-14^

In our study, among the benign soft tissue tumors, lobular capillary hemangiomas and dermatofibromas were frequently observed. Lobular capillary hemangiomas were also the most frequently seen benign soft tissue tumor in the study done by Sherpa and KC.^11^

Less number of the cases for the extensive variety of benign and malignant tumors is the limitation of this study. In the future, a similar study on a large number of cases from multiple centers following a uniform classification system could help find out the true incidence of skin tumors.

## CONCLUSIONS

Worldwide, a large number of studies have been reported pointing towards the increasing incidence of skin tumors and a similar trend is being seen in Nepal as evident from the multiple literature available in the matter.

The skin tumors have a wide and extensive categorical differentiation based on WHO and are thus required to be meticulously evaluated for understanding the pattern of distribution of the specific tumor type in different sexes and various age groups.

Our study concluded that the prevalence of skin tumors is increasing and the condition is seen more commonly in females. Benign skin tumors are commoner in young age groups whereas malignant skin tumors are more seen in elderly. This indicates the requirement of more screening facilities and extensive dissemination of information regarding the indolent lumps and bumps of the skin that are most often under looked by the patients. This can lead to early help seeking behavior and subsequently early detection and treatment for the condition.
